# The Development of an Arabic Weight-Loss App Akser Waznk: Qualitative Results

**DOI:** 10.2196/11785

**Published:** 2019-03-14

**Authors:** Ryan Alturki, Valerie Gay

**Affiliations:** 1 School of Electrical and Data Engineering Faculty of Engineering and Information Technology University of Technology Sydney Sydney Australia; 2 Department of Information Science College of Computers and Information Systems Umm Al-Qura University Mecca Saudi Arabia

**Keywords:** weight loss, mobile app, obesity, physical activity, smartphone, mHealth, motivation

## Abstract

**Background:**

Obesity and its related illnesses are a major health problem around the world. Saudi Arabia has one of the highest national obesity rates globally; however, it is not easy to intervene to prevent obesity and becoming overweight owing to Saudi Arabia’s cultural and social norms, and linguistic barriers. In recent years, there has been an exponential growth in the usage of smartphones and apps in Saudi Arabia. These could be used as a cost-effective tool to facilitate the delivery of behavior-modification interventions for obese and overweight people. There are a variety of health and fitness apps that claim to offer lifestyle-modification tools. However, these do not identify the motivational features required to overcome obesity, consider the evidence-based practices for weight management, or enhance the usability of apps by considering usability attributes.

**Objective:**

This study aimed to explore the opportunity and the need to develop an Arabic weight-loss app that provides localized content and addresses the issues with existing apps identified here. This study has explained the steps taken to design an Arabic weight-loss app that was developed to facilitate the adjustment of key nutritional and physical activities and behaviors, which considers the social and cultural norms of Saudi Arabia.

**Methods:**

Qualitative studies were conducted with 26 obese Saudi Arabians, who tested the level of usability of 2 weight-loss apps and then provided feedback and recommendations. The app Akser Waznk is an interactive, user-friendly app designed primarily for iPhones. It has several features intended to assist users to monitor and track their food consumption and physical activities. The app provides personalized diet and weight loss advice. Unique features such as *Let’s Walk* are designed to motivate users to walk more. An augmented reality function is implemented to provide information regarding fitness equipment, fruits, and vegetables. The app uses behavior-change techniques to increase activities and healthy behaviors and evidence-informed practices for weight-loss management. The Akser Waznk app considers user privacy and data security by applying a number of guidelines and procedures.

**Results:**

The development of the app took 26 months. In all, 7 experts (5 dietitians, and 2 physical activity professionals) evaluated the app’s contents. Moreover, 10 potential users (5 men and 5 women) tested the app’s level of usability, its features, and performance during a pilot study. They reported that the app’s design is interactive, and the motivational features are user-friendly.

**Conclusions:**

Mobile technology, such as mobile apps, has the potential to be an effective tool that facilitates the changing of unhealthy lifestyle behaviors within the Saudi community. To be successful, the target group, the usability, motivational features, and social and cultural norms must be considered.

## Introduction

### Background

Obesity refers to the process of storing extra energy in the body in the form of fat [1]. It is estimated that 39% of adults globally are deemed overweight and a total of 13% of the entire world’s population are obese [2]. Obesity is increasing at a rapid pace in Saudi Arabia, and it is believed that more than one-third (35.5%) of the population experiences this problem [3]. Obesity causes health problems and raises the risks of hypertension, cancer, and diabetes as well as cardiovascular and other diseases [4,5].

Some critical local factors have been identified that have caused the growth of this problem in Saudi Arabia. These include the increase in wealth and greater development in the country that has brought with it changes in lifestyle, with easier access to cars, international fast food chains, and the increased acceptance of processed food leading to a change in diet [6]. In addition, people lack nutritional knowledge, such as information about the calories in traditional and local foods [7]. Other significant factors are a lack of exercise and the country’s climate, which forces people to limit outdoor activities and stay indoors [8].

Furthermore, cultural aspects, beliefs, and restrictions, especially for women, could contribute to increasing the rate of being overweight and obese. In some local traditions, being overweight or even obese is considered a sign of high social status, beauty, prosperity, and fertility. In addition to this, women are encouraged to stay inside their homes and must be accompanied by their male guardians if they go out. Moreover, a male family member must agree and give permission to women to participate in physical activities [9]. Despite the widespread occurrence of obesity and overweight in Saudi Arabia, there is little treatment currently available. Gastric and bariatric surgery is the most popular way to reduce weight in Saudi Arabia as it is seen as the fastest and most effortless method; however, there are a number of risks and side effects related to it [10].

The use of smartphones and apps is common in Saudi Arabia, and the country is ranked as having the third largest global smartphone usage [11] penetration at 73% [12] as well as the largest global Twitter usage [13]. Smartphones can help people, especially women, to virtually interact publicly and socially when they would not normally be able to do so owing to cultural restrictions. Social media websites and apps, for example, Instagram, Facebook, and Twitter, are used by Saudi Arabians to start home-based businesses [14] and contribute to social solidarity [15]. Therefore, based on the growing ubiquity of the use of smartphones and apps, developing an Arabic app that can be used as a tool to treat and stop obesity in Saudi society is seen as vital.

### Previous Work

To ensure the developed app was suitable for its intended use, 4 significant aspects were examined. First, it was important to identify what features would help and encourage people with obesity to be active, change their lifestyle, and keep them motivated to overcome obesity. It was stated that goal-setting, monitoring, reminders, gamification, and rewards are features that can have a positive effect on overcoming obesity [16]. The second aspect to consider was the availability of effective weight loss apps, particularly in the Arabic language. For weight management, 13 evidence-informed practices are required, and the existing English [17,18] and Arabic [19] apps do not fulfill them. The third aspect involved consulting potential Saudi users of weight-loss apps regarding their use and requirements. After using such apps, these users reported language barriers, low usability levels, bad user experience, and cultural insensitivity. They recommend developing an Arabic weight loss app that is culturally sensitive [20-22]. The fourth aspect concerns the usability of the app. Recent studies found that, as of 2017, there were no Arabic weight-loss apps available that are designed with the aim of enhancing the level of usability of the app by addressing usability attributes to motivate users to lose weight [21,22]. The important usability attributes expected in any mobile app include effectiveness, satisfaction, efficiency, learnability, errors, and memorability as well as cognitive load [23,24]. The result from recent experimental usability testing for 2 weight-loss mobile apps built specifically to help Saudi users lose weight was that both apps had a low level of usability as they were developed without considering usability attributes and thus, users reported that both apps are difficult to use [21,22].

### Objectives

Given the information outlined in this section, the development of an Arabic weight-loss app that considers all 4 aspects is justified. This study aimed to describe the development process of the Akser Waznk app, the tools used to design the app, the obstacles faced by the designers, and the lessons learned through the process.

## Methods

### The Development

The Akser Waznk app is developed for iPhones. It is a user-friendly, interactive app that helps users to monitor and track their daily physical exercise and food consumption. It gives personalized advice for losing weight. The app took 26 months to develop, mainly owing to the need for professional information regarding motivational features to overcome obesity and the decision about what usability attributes should be considered while developing and designing the fitness mobile app. During the development of the app, we reviewed and explored the content of Arabic weight-loss apps and then chose 2 apps (Twazon and Aded Surat) and tested their level of usability with a group of 26 obese Saudi citizens (13 men and 13 women). We used their feedback to develop a fitness app which responded to their needs.

### What is Akser Waznk?

**Figure 1:**
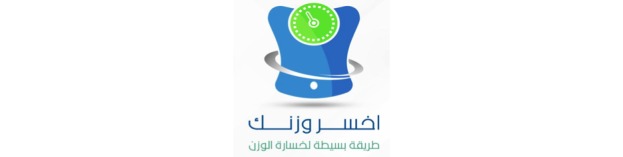
The logo and slogan of the Akser Waznk app.

The Arabic term *akser waznk* means *lose your weight*. This term was selected as the name for the designed app as it refers to the app’s main goal. The app aims to assist and motivate users to overcome obesity in a healthy way by helping individuals who are experiencing obesity to change their lifestyle. An initial focus group and participants from the local community were consulted to develop the app’s name, logo, and slogan (Figure 1).

**Figure 2:**
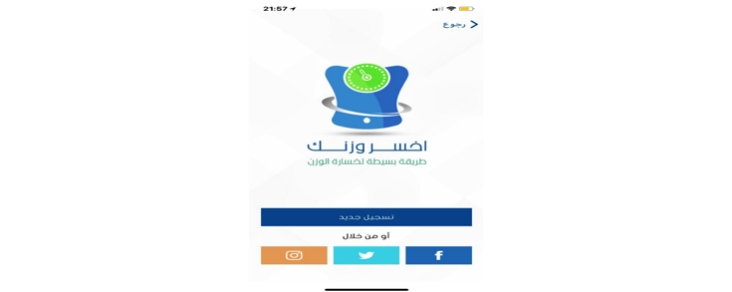
Membership and social network feature.

Users begin using the app with membership and social networking features (Figure 2). They have the option to create a new account by completing a 2-step registration process involving creating a user profile and inputting their information and body measurements or to sign in via their Facebook, Twitter, or Instagram account and then complete a 1-step registration process of inputting their information and body measurements. Creating a user profile requires users to input basic information such as their name and email address and to select a password. The information and body measurements step requires users to input their gender, age, height in centimeters, weight in kilograms, and, if known, the circumferences of their waist, buttocks, and hips.

In addition to these compulsory steps, an optional step—*health information* —can be completed, which requires users to provide information regarding 4 aspects: whether they use any medication permanently; if they have a chronic disease; whether they experience vitamin D deficiency; and if they are allergic to certain foods. Diet specialists will contact users in case 1 or more of the reported answers means the user requires a specific diet. First, they will provide users with a customized diet based on their needs and then help users to plan their future diets. This step was added based on recommendations from diet specialists as such factors affect users’ diet and weight-loss progress. After submitting the required information, users’ ideal weight and current body mass index (BMI) are displayed on the app screen.

There are a variety of different approaches that can be used to guide health promotion interventions. Social cognitive theory [25] forms the theoretical base of the Akser Waznk app because this theory addresses the importance of social systems relative to an individual’s behavior and considers the value of both self-efficacy and regulation. Moreover, this theory considers the dynamic interaction among personal, behavioral, and environmental factors, and the importance of observational learning that is established on observing others’ consequences and experience is confirmed.

The app contains numerous tools and features to address all the 13 evidence-informed practices for weight-loss interventions [17] (Table 1). The development of the app also considers the results and recommendations of both the experimental usability testing for 2 weight-loss apps [21,22] and the study that determines the motivational features to overcome obesity [16].

**Table 1:** The tools used in Akser Waznk to address evidence-informed practice for weight-loss management.

Practice	App information
Assessing and reaching an ideal weight	The waist circumference and BMI are calculated to assess each individual’s weight.
	The app provides a target weight and a date for individuals to use as their objective.
Healthy diet	On the basis of the users’ target weight, a daily calorie count required is calculated.
	Provides the recommended daily portions of food items and beverages.
	Provides the recommended daily amount of water.
	Generates and distinguishes recommendations based on the score of the weekly self-assessment.
	Helps users to understand and read labels on food products.
	Suggests substitutions of healthy foods for unhealthy food options.
	As an educational tool, the app shows users examples of nutritionally poor meals and diets to help them to plan their healthy meals.
	Users can plan healthy meals via a specific tool.
	The app will send notices if the calorie consumption and exercise deviate from the recommended level based on the score of the weekly self-assessment.
	The user’s consumption report helps to create a tailored healthy lifestyle circle.
Physical activity	The app suggests a minimum of 6 exercises from a list of physical exercises for the individual to do for a minimum of 45 min at least five times per week.
	The users are able to analyze their physical activity at the end of every week.
	The app provides videos and detailed information regarding all physical exercises in the app list for the users so that they execute the exercises in the proper manner.
	If physical activity is not achieved or the recommended level is not met, the app will send a reminder to the users.
	The *Let’s Walk* feature encourages the users to walk together every week.
	There is an added feature that provides directions so that users can walk to their nearest Mosque (place of worship).
	Gamification features encourage users to reach/achieve the daily count of steps goal, with the app donating money to charity every time the goal is met.
	Set an initial aim of 5000 steps, with this number gradually rising over time. A pedometer is offered to users so they can count the steps taken.
Self-monitoring and assessment	The app tracks the daily consumption of water, food (calories), performed exercise, and counts the daily steps taken.
	The users can perform the self-assessment regarding their physical exercise, food, and water consumption at the end of the week.
	The weight-loss tracker allows users to keep track of their weight-loss progress, offers their current weight in kilograms, and shows how they are meeting their weight-loss goal.
Social support	The users can use the built-in social network feature to communicate with other users, share their experiences, and provide useful tips and support.
	The users can have a 1-to 1-conversation with a qualified fitness trainer and diet specialists so that the users can raise any queries with professionals.
	The app can merge with social media platforms including Facebook.

### Assessing and Reaching the Ideal Weight

The app assesses users’ current weight based on the information that they input while creating their user profile. Lemmens et al’s [26] equations were used to determine users’ current BMI and ideal weight. The app allows users to set up a goal to lose either 0.5 or 1 kilogram per week [27], and users will be encouraged to have at least a moderate loss of their original weight (between 5% and 10%) because this amount of weight loss is significantly associated with significant changes in chronic disease risk [28]. The duration (in days or weeks) that is required to reach the ideal weight is calculated, allowing users to set realistic and appropriate goals. The Mifflin-St Jeor equation [29] for men and women was used to determine the daily calorie intake, as it was the most accurate and reliable calorie calculator equation available at the time of conducting this research [30].

### The Diet

**Figure 3:**
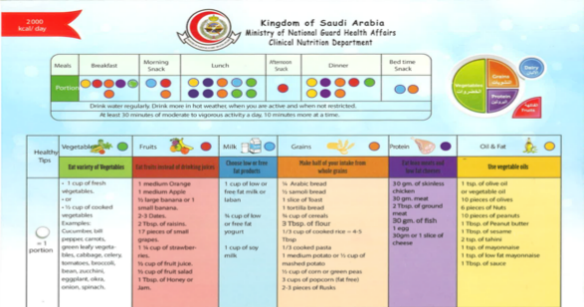
Clinical Nutrition Department’s diet template.

The app uses a diet template developed by the Clinical Nutrition Department at the Ministry of National Guard Health Affairs, Saudi Arabia, to help users to plan their daily meals (Figure 3). The template recommends users have 6 meals a day: breakfast, morning snack, lunch, afternoon snack, dinner, and bedtime snack. The template contains 6 food groups: vegetables, fruits, milk, grains, protein, and oil. The food portions for meals are determined according to the individual’s daily calorie needs. The app suggests meal times based on the recommendation of diet specialists.

**Figure 4:**
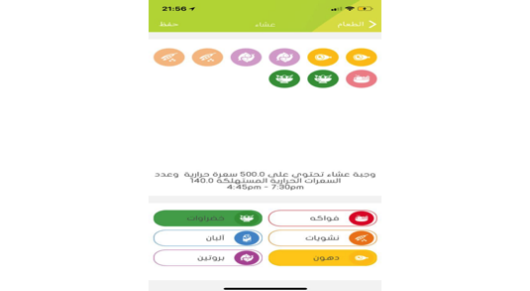
App implementing diet template.

**Figure 5:**
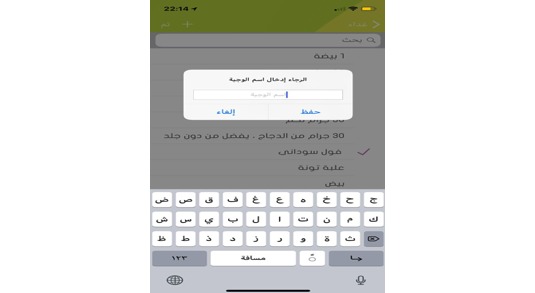
User suggests food item.

The app requires users to add the exact amount of their food portions from each food group for each meal. Users cannot exceed the daily requirement of calories or the calories recommended for each meal. The app has an interactive screen that makes planning or adding a meal easy for users (Figure 4). In the top part of the screen, users can see a number of circles that represent the different kinds of food groups that are displayed at the bottom of the screen. When users add a food item, its related food group circle turns on and, once users add all the required amount from a particular food group, the food group icon turns as well. If the user does not do this, a notification appears that asks users to follow the diet requirements to add a meal. In case the users consumed food that is not on the app’s food list, they can suggest their food items, and the app’s supporting team will address these foods to determine whether they should be added or not (Figure 5).

**Figure 6:**
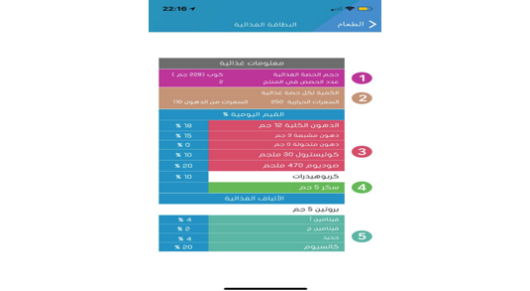
Description of how to read a food label.

**Figure 7:**
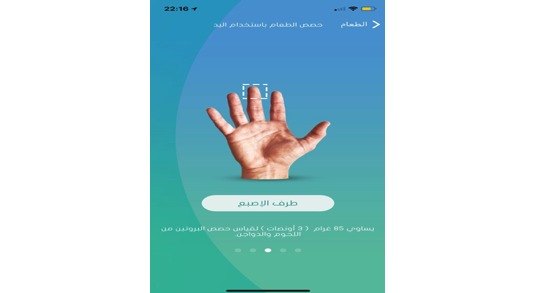
Determining food serving size by using hand.

The app also determines the recommend daily amount of water based on a specific equation [31] and describes the right way to read food labels (Figure 6) and how to determine a food serving size using the user’s hands (Figure 7).

### Customized Saudi Food Database

It was important to create a food composition caloric information database that included Saudi Arabian food varieties. The database uses the Clinical Nutrition Services Diet Manual from King Abdulaziz Medical City and Saudi food composition tables [32] as a base to offer both caloric information and serving size for more than 100 food items. However, as the caloric count of many traditional food items, for example, Shawarma and Mandee, were not available, we had to use common recipes from local restaurants and then calculate the nutrient values of different ingredients to determine the caloric count. The existing Saudi food databases use grams for measurement; yet, considering the traditional social norms, we were required to convert the quantity of food ingredients from measuring in grams to measuring with spoons, cups, or hands so that users could easily understand the quantities required. Furthermore, the app allows users to suggest new food items to be added to a specific database, with the app support team evaluating items before adding them to the official food database.

### Self-Assessment

There is a self-assessment feature in the app that allows users to keep track of their activities, performances, and progress during the week. Numerous studies have shown that eating a Mediterranean diet is helpful for reducing the obesity ratio [33,34]. Therefore, the app’s self-assessment technique is developed based on the Mediterranean diet assessment instruction [35]. However, as alcohol consumption is prohibited in the religion and culture of Saudi Arabia, alcohol was excluded. On the basis of both the Saudi Healthy Food Palm Guide [36] and Clinical Nutrition Department, Ministry of National Guard Health Affairs Guide, 5 extra questions were added, which cover an additional 4 aspects: the consumption of dairy, the consumption of whole grains, the consumption of water, and physical activity. The total score of the self-assessment is 18, with each question holding a value of 1 point (Multimedia Appendix 1). These questions help to analyze the level of consumption of different food items, and users’ responses are calculated to determine if their consumption is meeting the advised level or not.

**Figure 8:**
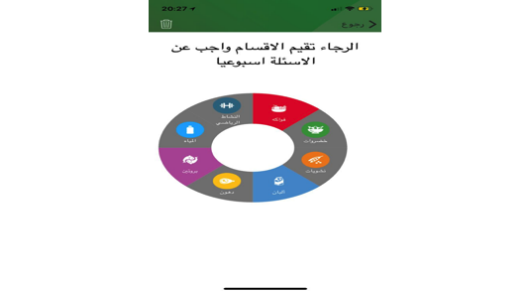
Graphic display regarding self-assessment results.

To make the results easy for users to understand, the results of the assessment will be presented in a graphic, user-friendly interface (Figure 8). When answers meet the recommended level, the graphic format will change from gray to a unique color in each aspect.

Users’ responses that do not meet the suggested level will be addressed by sending a notification to them that provides customized advice obtained from the Saudi Healthy Food Palm Guide [36] and international government health sources [37-39]. The app will also send a notification in the event that the performed activities, food and water consumption rates did not meet the recommended level according to the weekly self-assessment score.

### Physical Activities

**Figure 9:**
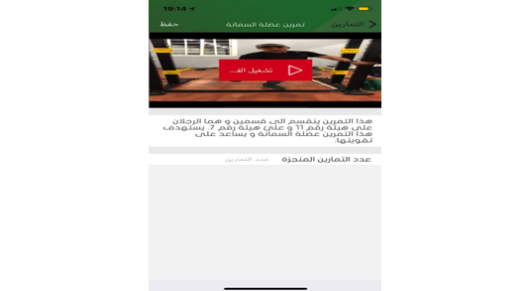
Physical exercise example.

The app’s physical exercise section was developed keeping in mind exercises that Saudi Arabians will be able to do. Exercises that are not available or possible for some users, for example, tennis and swimming, were substituted with different exercises that can be performed easily at home. This is a great benefit especially for female app users, who are not permitted to go outside unlike men. Each of the physical activities has information that describes the goal of the activity as well as a video to show the correct way it should be performed (Figure 9).

A qualified trainer will contact users once a month through the app’s chat feature to discuss the daily exercises. Users will also be able to talk to the trainer if they have a query. The app will recommend the user do 6 physical exercises from the provided exercises list for approximately 45 min at least 5 times a week.

**Figure 10:**
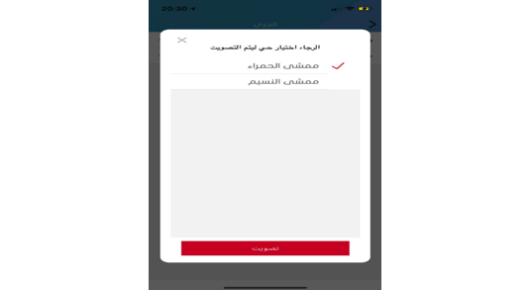
Voting screen.

The Akser Waznk app has 3 unique features that will inspire users to walk more. The first one is named *Let’s Walk*. With this feature, users can vote between 2 footpath locations to determine a place to gather with other users and walk as groups (Figure 10). The vote takes place weekly. At present, this feature works in 2 cities in Saudi Arabia (Makkah and Jeddah). The nominated places to be the starting point will be determined according to the footpath availability. The administration team will send a notification to users regarding the footpath that has been chosen each week based on the voting results.

**Figure 11:**
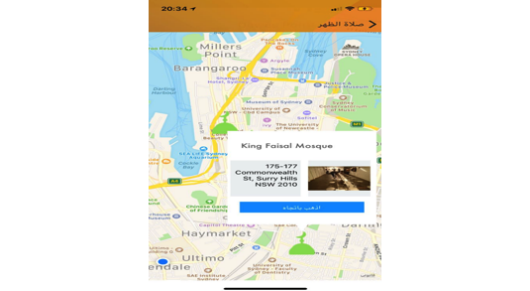
Mosques nearest location.

Saudi Arabia is a nation where more than 92% of the population believe and follow the Islamic religion [40]. In Islam, believers are required to pray 5 times in the day, and most men go to mosques to practice their prayers daily. Therefore, a second unique feature was developed, called *Walk to Mosque*. The app gives users the option to turn on notifications or alerts when it is a time of prayer that show the mosques nearest to the user’s location (Figure 11). If the users choose this option, the app will guide them. The app will give the users a choice of mosques so the user can walk to a more distant mosque if they want to increase their walking distance.

**Figure 12:**
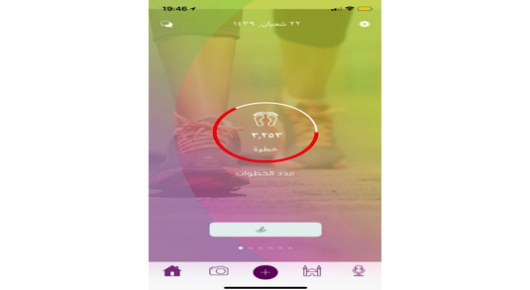
Tracking daily number of steps.

Several studies state that adding rewards to goal-setting features is a very useful way of increasing motivation and task performance [16] because they give a sense of achievement and satisfaction to the user and that leads to improved motivation and the achievement of fitness goals. Therefore, the gamification feature was implemented in this app. The app provides a pedometer that allows users to track their daily number of steps (Figure 12). Users have a daily goal of a certain number of steps to achieve and when it is achieved, they can donate a small amount to a charity. All the new users will have a credit of few cents at the beginning that can be used for the donation. Over time, the daily goal will then increase to the next level in increments of 500 steps until they reach a total of 10,000 steps.

### Self-Monitoring, Tracking, and Feedback

Self-monitoring is an essential part of the app because of the strong association between both physical exercises and a healthy diet with weight loss [41,42]. Thus, users will be able to self-monitor their daily intake of energy, for example, food (kCal), drinks and water consumption (in), the amount of physical exercise (out), and weight-progress tracking.

As it is important for the users to track and monitor their daily balance in the form of calories (in) versus exercise (out), the Akser Waznk app provides a customizable database for that purpose. By using this database, users have the ability to store their daily food intake and physical exercise performed and view their previous day’s history.

The home page has 6 horizontally scrolling sliding screens that provide information regarding the step count, exercise, meals, water consumption, weight-loss progress, and the summary of the day to allow easy access for tracking and goals. In addition to this, the app enables users to report their daily consumption of food and drinks and then calculates the number of calories eaten, so that it can inform users about the calories left to consume before the end of the day. Thus, the Akser Waznk app tracks the daily calorie consumption, steps taken, water consumption, and performed physical exercises.

**Figure 13:**
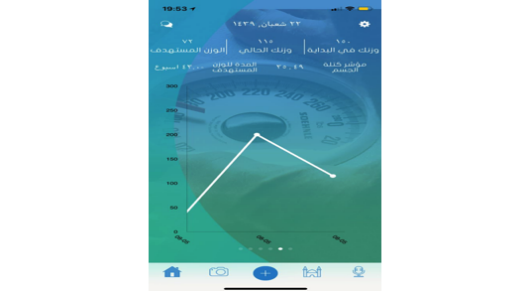
Weight loss progress.

Users cannot exceed the required amount of daily food and drink consumption (kCal) for weight loss, but if they do not consume the daily required amount of calories, drink enough water, or perform the recommend amount of physical exercise, the app will send a notification to remind them to engage more (see Multimedia Appendix 2 for a full list of the Akser Waznk app’s feedback and prompts). The app also allows users to monitor and track their weight development (Figure 13) by providing their starting weight, current weight, ideal weight, current BMI, and remaining duration (days or week) to reach their ideal weight. The app allows users to update their weight weekly and provides a graph that shows users their progress toward overcoming obesity.

### Social Communication

**Figure 14:**
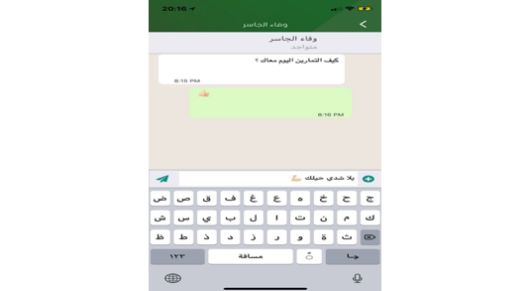
Chat between users.

The app allows users to connect to Web-based social media platforms and has a built-in chat feature (Figure 14) so that the users can share their experiences and tips with each other, which positively affects users’ health behavior [43]. This also enables users to get to know each other and then view each other’s progress, chat and post photos, and share them on several social media platforms, for example, Path, WhatsApp, and Twitter. This real-time communication allows users to get answers to various questions instantly, and users can answer queries by talking to each other. Many other weight-loss apps in both English [17,18] and Arabic [19] lack this feature, and the ability to do this will motivate the users of Akser Waznk. The decision to include this feature was based on usability testing and users’ recommendations to design the chat interface to be similar to other widely used messenger apps such as WhatsApp [21,22].

### Augmented Reality

**Figure 15:**
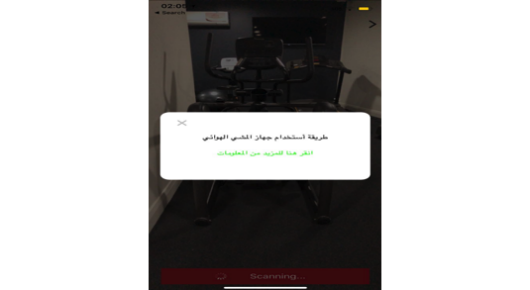
Scanning fitness equipment.

There are several technologies that have emerged recently and have been used widely in mobile apps as motivational tools, for example, augmented reality (AR) [44]. In AR, physical reality can become improved via the extra information that computers can produce in real time [45]. AR technology has been used in several mobile apps. In addition to this, it has been used in different fields, for example, education [46]. Such technology can even enable the better use of mobile devices for those with declining cognitive ability, such as people affected by Alzheimer’s disease [47]. As AR technology has proven that it can be used successfully in different fields by various groups of people and based on the users’ recommendations, the AR feature was implemented in the Akser Waznk app. The app allows users to scan fitness equipment to identify it and then the app will provide information regarding the benefit of using that type of equipment and the correct way for it to be used (Figure 15). The app identifies several fruits and vegetables common in Saudi Arabia and provides information regarding their average energy (kCal). For that purpose, a specific database was created to offer information for fitness equipment and food items.

### App Themes

After the app’s functionality and features, an app’s colors are arguably its most vital aspect. They assist an app’s users to see and interact with its contents, elements, and better understand actions [48]. Thus, they are seen as one of the most significant design features that strongly affect users’ evaluation and the perception of apps in general. However, selecting the color schema is a challenge, because it affects the level of usability of an app [48,49].

In this app, the traditional color scheme patterns (monochromatic, analogous, and complementary) were implemented. The app currently has 5 different themes that users can choose from. These themes were developed and tested with end users and experts from the initial focus group, and it was reported that they facilitate interaction with the app’s contents. According to the results of the usability testing and based on the users’ recommendations [21,22], an interactive design is seen as a vital tool that motivates users to keep using an app and hence improve the usability of an app. Therefore, a variety of app themes was presented.

**Figure 16:**
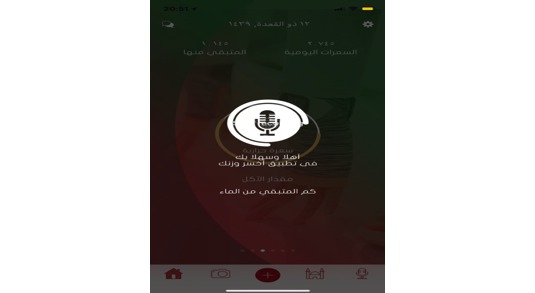
Voice commands.

### Voice Recognition

To ensure a user-friendly experience, the app supports voice commands for easy interaction [50]. This function allows users to add tasks or submit enquires using verbal commands (Figure 16). The app translates verbal command into written text on the screen and then performs what it is asked to do or answer the queries by either providing information on the screen or speaking to the user. At present, the app can do the following:

1. Navigate users to the *add meal* option.
2. Add the consumed amount of water.
3. Say the remaining amount of daily water consumption.
4. Say the consumed amount of water.
5. Say the remaining amount of daily calorie consumption.
6. Say the consumed amount of calories.

### Reminders

Many researchers have examined the effect of reminders on health in different settings and found that reminders are an effective motivational intervention [13,14]. Research [13] measures the impact of reminder features in apps as weight-loss intervention among obese individuals. The study explored the 6-month efficacy of a weight-loss intervention by mobile apps and found that intervention through reminders can produce modest weight loss. Therefore, the Akser Waznk app allows users to set a reminder, with users being able to determine the date, time, repetition, name, and the ringtone for a reminder.

### Behavior Change Techniques

The requirements and suggested features for an effective weight-loss app were based on both the feedback and recommendations gained from the target group [16,21,22] as well as all aspects of evidence-informed practices [17,18]. A number of existing weight-loss apps implement behavior change techniques (BCTs), and it is believed that BCTs can be helpful in increasing activities and healthy behaviors [51]. Therefore, these techniques are implemented in this app by using Michie’s taxonomy [52]. A total of 30 BCTs were incorporated into the following related goals: identity (1 code), goals and planning (4 codes), antecedents (3 codes), feedback and monitoring (4 codes), regulation (1 code), social support (3 codes), rewards and threats (3 codes), shaping knowledge (1 code), repetition and substitution (3 codes), natural consequences (2 codes), comparison of outcome (1 code), comparison of behavior (2 codes), and associations (2 codes). A reflection of the system prerequisites, BCTs, and relevant Akser Waznk app features are presented in the Multimedia Appendix 3.

### Data Privacy and Security

The Akser Waznk app provides accurate and personalized advice for weight loss through collection of users’ personal data. Personal data can be defined as information regarding an identified or identifiable natural person [53]. Due to the nature of the app and its use of private and personal information, the Akser Waznk app utilizes a number of the guidelines that were included in the European Commission’s Code of Conduct on privacy for mobile health apps [54] and the EU’s General Data Protection Regulation [53] to guarantee users’ security. These guidelines include purpose limitation, data minimization, and users’ consent. Upon signing up to the app, users are given a set of questions and an explanation of the purposes and method of the app before they consent to provide the necessary personal information including their age, gender, and weight. This forms an important dataset utilized to measure the user’s BMI, establish the target weight for the users, and provide the accurate diet plan. The app also requires users’ consent after creating an account and before actually using the app to access users’ health data via the iPhone Health option to retrieve data regarding steps and walking plus running distance. These data will be used to measure the daily walking steps and distance for users. In simple Arabic language, the app provides a detailed privacy policy outlining the purposes behind the data collected, permissions, and privacy statements as well as provision of necessary details of the app developers. This privacy policy for the Akser Waznk app can be found through either the Apple Store or through the app in the Setting option.

Moreover, to protect the users’ data, an advanced level of security procedure which is recommended by Martínez-Pérez et al [55] is performed, that is, encryption of the data. Encryption uses algorithms turning plain texts to unreadable text or jumbled code to ensure the security of the data and app. To decrypt this ciphertext, an encryption key is needed. Such a key is something which only authorized parties have in their possession [56]. The encryption protects 2 types of data: in-transit and at-rest data. In-transit data are data that are moving from 1 location to another, for example, when users input information on their mobile device, and the data are transferred to servers or databases. At-rest data refers to data that are not actively transferred and are instead stored, for example, in databases or clouds [57]. The Akser Waznk app considers both types of data and implements encryption techniques to ensure data protection.

## Results

### App Testing

During the testing phase for the Akser Waznk app, analysis was done by 2 groups: potential users and field experts living in Makkah and Jeddah, Saudi Arabia. Testers were given the app for testing, and they provided feedback and recommendations after using the app for 2 weeks.

### Expert Testing

The expert testing group comprised 7 health professionals in total: 5 dietitians (3 females and 2 males) and 2 male physical activity professionals. These testers evaluated the level of accuracy of the app by analyzing the app’s information, advice, and goals; and they confirmed that the information and advice provided by the app is accurate according to their professional experiences and knowledge. They had complete access to the app and its documentation. They stated that the Akser Waznk app meets all the required criteria, and its contents are effective and precise. The dietitians’ criteria include assessing users’ current weight, calculating current BMI, determining ideal weight, and allowing users to provide information regarding their health history and current status. In addition, this formula includes determining the daily calorie intake, providing 6 meals (3 meals and 3 snacks), encouraging users to eat from the 6 food groups, and determining the food portions for meals. Apart from this, the physical activity professionals’ criteria include providing a variety of exercises attainable for people who suffer from obesity. This section includes showing the correct way for the exercise to be done, explaining the goals of each exercise, guiding users to perform a minimum of 6 exercises at least 5 times a week, and encouraging daily walking.

In all, 4 of the dietitians responded positively with the app being able to determine the recommended calorie intake and food portions. A female dietitian reported:

The app determines the daily calorie intake and the food portions for meals. It does not allow users to add more calories within meals and ensure that users eat from all the different groups of foods. This is important that patients eat fruits, vegetables and all other food groups and not only focus on eating protein and grains. This is what I liked most of the app; it helps patients to have a healthy diet.

All the dietitians stated that knowing obese patients’ health history and current status is an important factor as it affects their diet and weight-loss progress. A male dietitian stated:

Users can report more information regarding their health status. Patients with chronic disease for example Diabetes do not eat some specific kinds of food. As a dietitian, it’s important to know our patients’ health status and history and the app helps in doing that.

The physical activity professionals liked the physical exercise section and how the app provides specific information regarding each kind of exercise such as the correct way to be performed and the goal of doing it. A physical activity professional pointed out that:

Through the app, people can see how exercises should be performed and know what is the benefit from them. I had several cases when people do exercises in the wrong way and then harm themselves and decides to stop practising.

Some enquiries were highlighted by the professionals regarding the use of voice commands as an input tool, the navigation to a mosque feature, and AR with the offline function and the gamification feature.

### Potential Users Testing

The potential users group comprised 10 obese Saudi Arabians. There were 5 men and 5 women in the group (Table 2).

They inputted their personal data and then followed the app’s goals, physical exercises, and recommendations as suggested so the testers could check the app’s features, level of usability, design satisfaction, user experience, and any other issues with the app’s performance.

#### Interview

After the trial, semistructured interviews that were audio-recorded were held with each of the potential users to gain their feedback and to respond to any queries. The testers were asked to provide feedback about what they liked and suggestions.

Visual aids such as word clouds were generated based on the data collected through extensive interviews [58]. Word clouds gather the amount and frequency of words and phrases used and display this through the size of the font [59]. In general, word clouds were utilized in social and commercial settings; however, they also have practical use in analysis because they provide rapid means to analyze textual data and reduce bias [60]. In the case of the interview data, 2 groups were formed with the answers (liked and suggestions), and 2 word clouds were generated showing common themes for each group.

**Table 2:** Demographic information about the potential users.

User	Gender	Age group (years)	Profession	iPhone model
1	Male	35 to 44	Self-employed	iPhone X
2	Male	25 to 34	Teacher at a high school	iPhone 7
3	Female	55 to 64	Retired	iPhone 6S
4	Female	25 to 34	Receptionist at a hospital	iPhone X
5	Male	45 to 54	Self-employed	iPhone 7
6	Female	Prefers not to say	Prefers not to say	iPhone X
7	Female	25 to 34	Accountant in a company	iPhone 7 Plus
8	Male	18 to 24	Student at university	iPhone 6
9	Female	45 to 54	Unemployed	iPhone 7 Plus
10	Male	25 to 34	Government employee	iPhone X

Table 3 displays the content of the original word cloud, and demonstrates that the majority of the users liked the app’s color schema and the variety of themes that they can choose from. User 4 said:

I like the colours of the app and what I liked more is the ability to change the whole themes.

They also liked the gamification features and how they were contributing socially while walking toward the daily counting-steps goal. User 7 stated:

Every time I walk, I remember that there are other people who will benefit from such walking. It is a good feeling and even encourage me to walk more and more.

The ability to self-monitor weight-loss progress and the ease of tracking daily activity and the consumption of both food and water were appreciated by the majority of users. User 1 reported:

All the tracking screens are in the main screen, I just scroll right and left, and all the information is there. It is great to know how many calories I had and how much is left. The same thing for the water consumption as well. What I liked most is the weight progress screen. When I updated my weight, I can see a chart that shows me the exact date of updating my weight and even in the screen there is my start, current and goal weight.

The AR feature and how it can help to provide information regarding fitness equipment was also appreciated. User 6 pointed out:

It is great and easy to use, this is the first app I used that has such a feature.

### Suggestions for Future Versions of the App

Table 4 shows potential users’ most-mentioned words regarding the suggestions to improve the app. User 4 suggested that it would be good for users to be able to customize the color schema as well as the app’s themes. In addition to this, Users 1 and 5 wanted to receive notifications to remind them about meal times. Furthermore, User 3 mentioned that it would be good to have a tutorial on how to use the app. Another suggestion by Users 1, 4, 5, 6, and 10 was to make the app integrate with fitness and watch trackers such as Apple watches and Fitbits. Users 1, 3, 4, and 6 suggested that this app should have sponsors or that users should have the ability to donate to charities when they have achieved their daily steps goal.

**Table 3:** What potential users liked about the app.

Most common words	Frequency of use
Colors	8
Tracking	7
Goal weight	5
Donate	3
Loss	3
Augmented reality	2
Screen	1
Progress	1
Calories	1
Easy	1

**Table 4:** Potential users’ suggestions for the app.

Most common words	Frequency of use
Apple Watch	7
Sponsors	5
Notifications	4
Themes	3
Colors	3
Remind	2
Times	2
Meal	2
Tutorial	1
Customize	1

## Discussion

### Principal Findings

The Akser Waznk app aims to facilitate the change of unhealthy lifestyle behaviors within the Saudi community by identifying and implementing the motivational features [16], considering both the Saudi social and cultural norms [20-22] and usability attributes [21,22]. As stated in previous studies, other Arabic weight-loss apps do not comply with the needed evidence-informed practices for weight-loss management [19,21], and the research and development of Akser Waznk app aim to effectively contribute to such an issue. The Akser Waznk app is different than other Arabic weight-loss apps in the following ways:

1. End-users and experts in the field of exercise and diets helped design the app, with their requirements, feedback, and recommendations incorporated into the beta version.
2. The app is in line with all 13 evidence-informed practices for weight-loss management.
3. Social network access for interacting with other app users is provided.
4. The app contains calorie counts of Saudi foods.
5. The app’s design considers usability attributes.
6. The app considers the social and cultural norms of Saudi citizens.
7. Users can view the history of their calorie consumption and exertion via a customized database.
8. The app has a gamification feature to encourage users to walk more.
9. The app provides information regarding the correct use of a fitness equipment via an AR feature.
10. The app supports voice commands.
11. The app encourages group-walking via the *Let’s Walk* feature.
12. The app can guide users to mosques near their location.

A group of 7 experts (5 dietitians and 2 physical activity professionals) and 10 potential users (5 men and 5 women) from the Saudi community tested the Akser Waznk app to evaluate the app for usability, performance, and motivational features. The app was also evaluated in terms of how accurate the information it provides is. The testing found that the app assisted numerous potential users to understand their weight-loss goals and techniques. Moreover, the testers reported that the app’s design is esthetically pleasing, and the majority of motivational features are user-friendly.

Following feedback from the testers, new users will be guided via a tutorial consisting of pop-up messages within the app for the first time that identifies the most significant points and steps. This will better clarify the app’s functions and features. Notifications of meal-times and the ability to customize the color schema and the app’s themes are features which will be worked on and integrated in a future version of the app. Moreover, the food database bank will be constantly updated to provide the nutritional data of additional foods.

### Limitations

The current Akser Waznk app has some limitations. It is not available on smartphone platforms other than iPhone, for example Android and BlackBerry. The app also currently does not support virtual reality features and is not integrated with smartwatches, such as Apple watches, or fitness trackers, for example, Fitbit. It does not support the barcode-scanning feature for food items, and the app cannot work offline. There is no sponsor for the gamification feature, and the app does not allow users to donate to charities directly. In addition to this, the response time for the health information step might take up to a week as there are just 5 diet specialists participating as consultants. However, finding possible solutions to these limitations in the near future and updating the app regularly will further help to motivate and keep users engaged.

### Strengths

The Akser Waznk app is different from its counterparts in a number of ways. It is built on all of the 13 evidence-informed practices for weight-loss management, and it addresses the initial focus group’s feedback and recommendations, which allows the app to meet the specific requirements of obese Saudi people via a localized and tailored method. The Akser Waznk app is currently the only app in Saudi Arabia that has gamification, AR, voice command features, and it is the only app which encourages weekly walking groups via the *Let’s Walk* feature and daily walking via *Walk to Mosque* feature. The app offers local Saudi household measurement units, such as cups and spoons for local and traditional foods, which makes it easier for users to manage their daily portion control. The daily physical exercise suggested by the app meets the social and cultural norms of Saudis and suits users’ physical status. The app provides a social media platform especially for the app’s users, which allows them to share information and support and motivate each other. Finally, advice and recommendations to avoid specific foods and increase the consumption of others are sent by notification to users based on the results of their weekly self-assessments.

### Potential Impact

Lacking nutritional knowledge, exercise, limitation for outdoor activities owing to Saudi Arabia’s climate and restrictions especially for women are some of the critical local factors that contribute to increasing rates of obesity. Smartphones are becoming very popular in the country, and this is changing many dynamics of the conservative society. The Akser Waznk app aims to help people who suffer from obesity to lose weight and improve their overall lifestyle. The app is a low-cost alternative to traditional personal behavioral weight-loss programs as it considers the needs of Saudi obese users, unique living habits of the country, and provides several unique features that motivate users to monitor and track their food consumption and increase their physical activity. On the basis of the results of the experts’ evaluation, potential users’ usability testing, and feedback, it is believed that the usage of Akser Waznk app will decrease the obesity rate in the country.

Motivational features such as *Let’s Walk* will encourage people to gather together to walk. As more than 92% of the Saudi population believe and follow the Islamic religion, the majority of people go to mosques 5 times a day. The *Walk to Mosque* feature will, therefore, contribute, providing attainable exercise with the potential to make it communal. It will encourage and guide users by giving them a choice of mosques near their location, and users will have the ability to choose to walk to a more distant mosque if they want to increase their walking distance. The app also provides a pedometer, allowing users to track their daily steps and when users reach their daily goal of walking, they can donate a small amount to a charity. Users will be able to share their achievement with their peers within the built-in chat feature or with their friends on other social media platforms which will positively affect users’ health behavior. The app will also encourage users to set up a goal to lose either 0.5 or 1 kilogram per week and will determine the daily calorie intake and the duration (in days or weeks) to reach their ideal weight. Including local food varieties and providing their ingredients and measurement in an easy way to be understood, such as spoons, cups, or hands, is another aspect that can motivate users to follow their diets. All of these and other features with the ease of use of the app will positively contribute in the acceptance of the app among the target group and help to decrease the rate of obesity in Saudi Arabia.

### Next Steps

To better determine the usability level of the app and improve it, a quantitative study will be conducted with a group of 26 obese Saudi citizens (13 men and 13 women) from the cities of Jeddah and Makkah. Also, a 3-month pre- and postintervention study with the group will be done to determine the effectiveness of the app on weight-loss management. Users who lose at least 2.5% of their body weight will be the main endpoint for this study. In addition to this, developing the app in other popular mobile phone platforms, for example, Android, and integrating the app with smartwatches and fitness trackers are other objectives in the near future.

## Appendix

Multimedia Appendix 1 Lifestyle self-assessment questions.

Multimedia Appendix 2 Full list of the Akser Waznk app’s feedback and prompts.

Multimedia Appendix 3 A reflection of the system prerequisites, behavior change technique and relevant Akser Waznk app features.

Multimedia Appendix 4 Informed Consent Form and approval.

## Abbreviations

AR: Augmented Reality
BCTs: behavior change techniques
BMI: body mass index
