# Supplementary material for: The Development of an Arabic Weight-Loss App Akser Waznk: Qualitative Results
Source: JMIR Form Res. 2019 Mar 14;3(1):e11785. doi: 10.2196/11785 (PMC6437613; doi:10.2196/11785)
Supplement: Multimedia Appendix 1 [file formative_v3i1e11785_app1.pdf]

Multimedia Appendix 1: Lifestyle self-assessment questions

Aspect	Question
Vegetables	Are you eating two or more servings (of 200g each) of vegetables every day?
	Are you eating pasta, vegetable or rice dishes with garlic, tomato, leek or onion two or more times per week?
Fruits	Are you eating three or more servings of fruit (of 80g each) every day?
Oils	Do you use olive oil as the main culinary fat?
	Do you use four or more tablespoons of olive oil every day?
	Are you eating less than one serving (12g) of butter, margarine, or cream every day?
Sugar	Are you consuming less than one serving (330ml) of sweet or sugar-sweetened carbonated beverages every day?
	Are you eating less than three servings of commercial sweets/pastries every week?
Meat and beans	Are you eating less than one serving (100–150g) of red meat/hamburgers/other meat products every day?
	Are you consuming three or more servings (of 150g) of legumes every week?
	Are you eating three or more servings of fish (100–150g) or seafood (200g) every week?
	Are you consuming less than one serving (30g) of nuts every week?
	Are you eating chicken, turkey or rabbit routinely instead of veal, hamburger or sausage?
Dairy	Are you eating two or less servings of dairy products every day? (One serving is one cup of milk or yogurt, three slices of processed cheese slices)
	Are you consuming low fat or skimmed milk products in place of full fat?
Bread and cereal	Did you eat wholegrains in place of refined grains?
Water	Did you consume the recommended water quantity per day?
Physical activities	Did you do any kind of physical activity for 45 minutes five or more times per week or more?
