# Supplementary material for: The Development of an Arabic Weight-Loss App Akser Waznk: Qualitative Results
Source: JMIR Form Res. 2019 Mar 14;3(1):e11785. doi: 10.2196/11785 (PMC6437613; doi:10.2196/11785)
Supplement: Multimedia Appendix 2 [file formative_v3i1e11785_app2.pdf]

Multimedia Appendix 2: Full list of the Akser Waznk app's feedback and prompts

**Category**		**Kind of feedback**
Physical activity	Daily gauge	Visual feedback<br><br>Homepage: The 'physical activity' circle gauge completes itself and turns purple when users perform the required amount of daily exercises.
	User did not perform the required amount of exercise	Visual feedback<br><br>Homepage: The physical activity's circle gauge remains partially completed and part of it remains grey.
Step counter	Daily gauge	Visual feedback<br><br>Homepage: The 'step counter' circle gauge completes itself and turns red when users walk the required number of daily steps. In the beginning it will be 5,000 steps and then it gradually increases by 500 steps until it reaches 10,000 steps.
	A user walked 1,500 steps or fewer	Popup message: "It is not easy to reach 5,000 steps for the first time. Try to walk more tomorrow."
	A user walked between 1,501 and 2,499 steps	Popup message: "You did great, you almost reached halfway. Keep it up!"
	A user walked between 2,500 and 3,500 steps	Popup message: "Good! You walked more than halfway. You can make it tomorrow."
	A user walked between 3,501 and 4,999 steps	Popup message: "Amazing! You almost reached your goal. Tomorrow is the day."
	A user walked between 5,000 and 7,499 steps	Popup message: "Congratulations! You reached your goal."
	A user walked between 7,500 and 9,999 steps	Popup message: "Excellent! You can do this every day."
	A user walked 10,000 steps or more	Popup message: "Unbelievable! You must be proud."
Food consumption	Daily gauge	Visual feedback<br><br>Homepage: The user's daily calorie consumption and the calories remaining are calculated and showed as numbers. The food intake's circle gauge completes itself and turns yellow when users consume the required amount of daily calories.<br><br>Food's database: The user's daily calorie consumption and the calories remaining are calculated and showed as numbers.

	User did not consume the required amount of calories	Visual feedback<br><br>Homepage: The food intake's circle gauge remains partially completed and part of it remains grey.
Water consumption	Daily gauge	Visual and spoken feedback<br><br>Homepage: The user's daily water consumption and the amount remaining are calculated and showed as numbers. The water consumption's circle gauge completes itself and turns blue when users drink the required amount of daily water.<br><br>Homepage: The app displays the user's water consumption so far and the remaining amount to be drunk that day.
	User did not drink the required amount of water	Visual feedback<br><br>Homepage: The water consumption's circle gauge remains partially completed and part of it remains grey.
Donate to charity	After a donation is achieved	Popup messages:<br><br>"You are helping to make a better world"<br><br>"You have made a difference"<br><br>"We are grateful for your generosity"<br><br>"We appreciate it"<br><br>"Thank you so much"
Self-assessment	When the self-assessment is done	Visual feedback<br><br>Self-assessment page: Each section in the circle turns to a different colour when users answer correctly; otherwise, sections will remain grey.
	When users complete the self-assessment successfully	Popup message: "Well done! You are following a healthy lifestyle."
	When users complete the self-assessment unsuccessfully	Popup message: "Oh! We encourage you to avoid unhealthy habits."
	Remind users to do the self-assessment	Popup message: The apps send a notification to remind users saying "Please do the self-assessment"
Goal setting	Duration to reach ideal weight	Visual feedback<br><br>Homepage: The duration to reach the ideal weight is showed in days or weeks.<br><br>Homepage: BMI value is displayed, and it is updated when users update their weight.

	Information about updated user's BMI	Visual feedback<br><br>Settings page: Information on how to calculate the user's BMI
	User's ideal weight information	Visual feedback<br><br>Settings page: Information on how to calculate the ideal weight value for male and female.
Self-monitoring and tracking	Reminds user to update weight every week	Popup message: The app sends a reminder notification to users saying "Please update your weight"
Social notifications	New message	Visual feedback<br><br>Chatting page: Users see messages<br><br>Popup message: "New message from (name)"
Alarm notification	Reminder	Popup message: The app notifies users when the alarm goes off (the user names the alarm themselves).
Advice for a healthier lifestyle	Daily general health advice	Popup message: The supporting team sends daily general health advice. For example:<br><br>"Avoid consuming soft drinks"<br><br>"Eat fruits and vegetables every day"
	Advice based on the weekly self-assessment score	Popup message: A tailored notification is sent to the user from an archive of advice regarding the eight topics of the self-assessment. For example:<br><br>"Use low or fat free dairy products instead of full fat"<br><br>"Use olives oil with the green salad"
