# Supplementary material for: The Development of an Arabic Weight-Loss App Akser Waznk: Qualitative Results
Source: JMIR Form Res. 2019 Mar 14;3(1):e11785. doi: 10.2196/11785 (PMC6437613; doi:10.2196/11785)
Supplement: Multimedia Appendix 3 [file formative_v3i1e11785_app3.pdf]

Multimedia Appendix 3: A reflection of the system prerequisites, BCTs and relevant Akser Waznk app features

System requirements	BCT	Associated features in the app
Arabic language and culturally sensitive.	**Shaping knowledge (4.1):** Advise or suggest how to execute the behaviour.	Offers all information in the Arabic language including numbers and texts in a dialect with an easy, traditional and sensitive approach. This permits users to comprehend the app's advice and lose weight.
Improving motivation tools via delivering qualified guidance and optimistic strengthening.	**Information about other users' approval (6.3):** Deliver information about other users' perception about the behaviour.	Other users' view of the posts on the social network will help to deliver inspiration and advice to users. For instance, receiving "likes" for what you have for dinner.
	**Identification of self as role model (13.1):** Show how one's own behaviour can set a strong example for one's followers.	The users are encouraged to think of themselves as a role model for their friends and family members.
	**Credible source (9.1):** Offer a visible or oral mode of communication from a reliable source against or in favour of the behaviour.	Professional guidance and optimistic strengthening is achieved through the app by informing the users about their progress, objectives and encouraging them to keep working via sending messages. Because this is an evidence-based app, the messages and sources are deemed as reliable.
	**Instruction on how to perform a behaviour (4.1):** Advice or suggest about how to execute the behaviour.	Expert guidance for users to apply evidence-informed practices is provided via the app's notifications and tips. The users are also given valuable information regarding the impacts of unhealthy food choices and abdominal obesity.
	**Information about health consequences (5.1):** Deliver valuable information regarding the impact on health as a result of the behaviour.	Guidance is provided to users with the aim of encouraging them to eating healthy food and prevent them from consuming high calorie foods regularly. For instance, instead of eating a cake as a snack at work, instead eat fruit such as a banana or apple.
	**Remove access to the reward (7.4):** Guidance is provided to users	Guidance is provided to substitute a behaviour that

	so that they avoid the undesired behaviour.	contributes to overweight and obesity, such as eating unhealthy foods, with healthy food products.
	**Behaviour substitution (8.2):** Rapid replacement of undesired behaviour with a neutral or wanted one.	Expert guidance to reshape living circumstances and move towards making more healthy decisions through the app.
	**Restructuring the physical environment (12.1):** Suggest changes in the physical environment with the purpose of preventing undesired behaviour and enhancing the performance of the desired behaviour.	Guidance is provided to the users to reshape their social conditions by participating in weight-loss programs, communicating with others and being involved in physical activity, for example playing football with friends or family members or other activities that can contribute to weight loss.
	**Restructuring the social environment (12.2):** Suggest changes in the social environment with the purpose of preventing undesired behaviour and enhancing the performance of the desired behaviour.	Guidance to replace unhealthy eating habits with new healthy eating habits and behaviour.
	**Habit reversal (8.4):** Rapid recurrence and rehearsal of substitute behaviour in place of an undesired habitual conduct.	Built-in chat platform where users can exchange their success and failures and get inspiration from those who have achieved results in order to reach their goals.
Physical activity and dietary tools	**Goal-setting behaviour (1.1):** Resolve or decide on a goal with regard to the behaviour to be attained.	The users' BMI and weight loss goals are calculated after users provide their information. The app then determines users' ideal weight and sets consumption strategies and target dates for the users to achieve it.
	**Goal-setting outcome (1.3):** Resolve or decide on a goal with regard to the positive result of desired behaviour.	The app sets a target to reduce 0.5 to 1 kilogram every week as a consequence of following the app's instructions and recommendations.
	**Action planning (1.4):** Rapid detailed planning of behaviour's performance.	The app sends regular notifications to users so they do not forget to exercise, walk and consume water and food as recommended. The app also sets a calorie target for each day and notifies the

		users when they reach the limit.
	**Discrepancy between current behaviour and goal (1.6):** Draw attention to inconsistencies between the formerly set action plans, conclusion goals or behavioural goals with the present behaviour.	Graphical features are added in the app that reminds the users if they are deviating from their weight-loss guidelines.
	**Graded tasks (8.7):** The tasks should begin at an easy level and then slowly become more difficult but also achievable at the same time.	The app increases the number of steps and the amount of exercise over time through the graded weight-loss goals.
	**Conserving mental resources (11.3):** Suggestions to assist in behaviour change by reducing pressure on mental resources.	The app teaches users to understand nutritional labels to reduce the need to search for the calorie count every time. The food calorie guide in the app also helps users by providing different calorie counters. The app also promotes doing exercise and uses a step counter to enhance physical activities.
	**Body changes (12.6):** Facilitating behaviour change via altering body structure, functioning or support directly.	When behaviours are performed, the messages are automatically reduced and the prompt settings are set accordingly.
	**Reduce prompts/cues (7.3):** To perform the behaviour, the gradual removal of prompts is implemented.	
Tailored feedback and information	**Feedback on behaviour (2.2):** Fulfil and supervise evaluative or informative feedback on users' performance.	The feedback is given to users with regard to their daily or weekly data archives.
	**Self-monitoring of behaviour (2.3):** Create a technique for the user to record and observe their behaviours as a segment of behavioural change tactics.	The saved data is used to evaluate and observe users' behaviour.
	**Self-monitoring of outcomes of behaviour (2.4):** Create a process for the user to observe and record the results of their behaviours as a segment of behavioural change tactics.	The app suggests users record their weight once a week and then advices users to track via an updated app setting.
	**Feedback on outcomes of behaviour (2.7):** The users should be updated about their weight-loss reduction status when they have completed their exercises and updated their weight.	The app provides information to users about the duration in days or weeks they have spent doing physical exercise and updated their weight.

	**Reduce prompts/cues (7.3):** To perform the behaviour, the gradual removal of the prompts is implemented.	After every weekend assessment, the feedback messages are adjusted. The messages are reduced when the conduct is achieved and are tailored according to the users.
	**Awareness (2.5):** The app provides detailed information regarding particular points and helps users with awareness.	There are videos and tutorials in the app that show the proper ways to do different exercises.
User-friendly interface	**Shaping knowledge (4.1):** Provide advice or suggestions as to how to execute the behaviour.	The app's user-friendly interfaces comes with a designed built-in BCTs to help them understand the goals they must achieve for weight loss.
Social networking and support	**Social competition (6.2):** Highlight the performance of other users and allow users to compare it with their own performance	Social networking helps users to motivate themselves and improve their own performance. The users would be able to see the percentage of users achieving their goals.
	**Social support - unspecified (3.1):** Deliver social support when conduct is achieved.	The built-in social feature allows different users to share their stories and suggestions and to improve their performances via the app's social network or other social networks, for example Facebook and Twitter.
	**Social support - practical (3.2)**: Deliver practical help to achieve the desired conduct.	Users can share their experiences in private windows by sharing their photos, weight-loss data and other valuable information to help them to provide and get emotional and practical support.
	**Social support - emotional (3.3):** Deliver emotional support to achieve the conduct.	
Metafictional toll rewards	**Non-specific reward (10.3):** The reward should only be provided if progress is seen in achieving the behaviour.	As the users attain their goals, the app congratulates the users through notifications which help to boost their motivation. The app also shows a coloured circle when all the goals are met over a week.
	**Social reward (10.4):** If the daily step count is achieved then a social reward is provided.	The users have the option of donating a small amount of money to charity when they achieve their goal.
	**Self-reward (10.9):** If the daily step count is achieved then a self-reward is provided.	The app generates a thank-you message each time a user donates to a charity.

Self-assessment	**Self-assessment of affective consequences (5.4):** Create a procedure for the users to self-assess themselves regularly.	The app encourages the users to complete their weekly self-assessment option and also provides the results.
