# Supplementary material for: The Development of an Arabic Weight-Loss App Akser Waznk: Qualitative Results
Source: JMIR Form Res. 2019 Mar 14;3(1):e11785. doi: 10.2196/11785 (PMC6437613; doi:10.2196/11785)
Supplement: Multimedia Appendix 4 [file formative_v3i1e11785_app4.pdf]

UNIVERSITY OF TECHNOLOGY SYDNEY

Human Research Ethics Committee
Ethics Secretariat
C/O Research and Innovation Office
15 Broadway, Ultimo NSW 2007

T: +61 2 9514 9681
Research.Ethics@uts.edu.au

PO Box 123
Broadway
NSW 2007 Australia
www.uts.edu.au

UTS CRICOS PROVIDER CODE 00099F

21st September 2016

Associate Professor Valerie Carole Jeanne Gay
School of Electrical and Data Engineering
UNIVERSITY OF TECHNOLOGY SYDNEY

Dear Valerie,

UTS HREC ETH16-0833 – Associate Professor Valerie Carole Jeanne Gay; Mr Ryan Mansor A Alturki; – "Improving Mobile Fitness Application Usability Guidelines to Help Users Reach Their Health and Fitness Goals, Case Study: Obesity Apps in Saudi Arabia"

Thank you for your response to the Committee's comments. Your response satisfactorily addresses the concerns and questions raised by the Committee who agreed that the application now meets the requirements of the NHMRC National Statement on Ethical Conduct in Human Research (2007). I am pleased to inform you that ethics approval is now granted.

Your approval number is UTS HREC REF NO. ETH16-0833.
Approval will be for a period of five (5) years from the date of this correspondence subject to the provision of annual reports. Your approval number must be included in all participant material and advertisements. Any advertisements on the UTS Staff Connect without an approval number will be removed.

Please note that the ethical conduct of research is an on-going process. The *National Statement on Ethical Conduct in Research Involving Humans* requires us to obtain a report about the progress of the research, and in particular about any changes to the research which may have ethical implications. This report form must be completed at least annually, and at the end of the project (if it takes more than a year). The Ethics Secretariat will contact you when it is time to complete your first report.

I also refer you to the AVCC guidelines relating to the storage of data, which require that data be kept for a minimum of 5 years after publication of research. However, in NSW, longer retention requirements are required for research on human subjects with potential long-term effects, research with long-term environmental effects, or research considered of national or international significance, importance, or controversy. If the data from this research project falls into one of these categories, contact University Records for advice on long-term retention.

If you have any queries about your ethics clearance, or require any amendments to your research in the future, please do not hesitate to contact the Ethics Secretariat at the Research and Innovation Office, on 02 9514 9772.

Yours sincerely,

Professor Marion Haas
Chairperson
UTS Human Research Ethics Committee
C/- Research & Innovation Office
University of Technology, Sydney
E: Research.Ethics@uts.edu.au

UTS
UNIVERSITY OF TECHNOLOGY SYDNEY

INFORMED CONSENT FORM

Improving Mobile Fitness Application Usability Guidelines to Help Users Reach Their Health and Fitness Goals, Case Study: Obesity Apps in Saudi Arabia

Education UTS HREC Approval No. ETH16-0833

I, ____________________, agree to participate in the research project "Improving Mobile Fitness Application Usability Guidelines to Help Users Reach Their Health and Fitness Goals, Case Study: Obesity Apps in Saudi Arabia" UTS HREC Approval No. ETH16-0833 being conducted by Ryan Alturki and Valerie Gay C/O PO Box 123 Broadway, NSW 2007; +61 2-9514-7605.

I understand that the purpose of this study is to identify how to improve "usability" and user experience while using mobile fitness applications. The aim of this research is to make mobile fitness applications more usable and to help people who suffer from obesity to stay motivated.

I understand that I have been asked to participate in this research workshop because I am in a position to provide informed opinions regarding a mobile fitness application and that my participation in the course of this research workshop will involve the testing of the application. Firstly, I will fill out a brief questionnaire, which will take me approximately 5 minutes to complete. I will then test the mobile fitness application called "Akser Waznk". This should take me approximately 40 minutes to complete and the screen of my mobile device will be recorded during this time. During these 40 minutes, I understand that the researcher will be observing my body language as well as my facial expressions. Finally, I will participate in a 15 minutes semi-structured interview that will be audio recorded.

While no harm is intended, invasion of privacy, embarrassment or distress may arise unexpectedly. I will be reporting and discussing my experience which will require some level of personal disclosure. While the testing is anonymous, there is a small chance that I may feel privately embarrassed if I do not manage to perform a task correctly the first time. I am aware that I will not be judged on my performance. I understand the researcher is only seeking my personal opinion on the app and its usability.

I am aware that participation in this research is voluntary and I am free to withdraw from any further participation in this research at any time without any consequences.

I agree to be:
☐ Audio recorded
☐ Video recorded

I agree to keep confidential all information including all conversations and discussions, materials and methods provided to me by the UTS research team.

I agree that the research data gathered from this project may be published in a form that:
☐ Does not identify me in any way

I am aware that I can contact Ryan Alturki via his email address, (ryan.m.alturki@student.uts.edu.au) or via mobile (+966 555511160), Valerie Gay via her email address, (valerie.gay@uts.edu.au) or via phone (+61 2 95144645) or the local independent contact person, Dr. Abdulrahman Alaryni, via his email address, (dr.alaryni@hotmail.com) or via mobile (+966 554546535) if I have any concerns about the research. I also understand that I am free to withdraw my participation from this research project at any time I wish, without consequences, and without giving a reason.

I agree that Ryan Alturki and Valerie Gay have answered all my questions fully and clearly.

____ ____/____/____
Name and Signature (participant) Date

____ ____/____/____
Name and Signature (researcher or delegate) Date

NOTE:
This study has been approved by the University of Technology Sydney Human Research Ethics Committee (UTS HREC). If you have any concerns or complaints about any aspect of the conduct of this research, please contact the Ethics Secretariat on ph.: +61 2 9514 2478 or email: Research.Ethics@uts.edu.au, and quote the UTS HREC reference number. Any matter raised will be treated confidentially, investigated and you will be informed of the outcome.
